# Corticosterone Induces Rapid Spinogenesis via Synaptic Glucocorticoid Receptors and Kinase Networks in Hippocampus

**DOI:** 10.1371/journal.pone.0034124

**Published:** 2012-04-11

**Authors:** Yoshimasa Komatsuzaki, Yusuke Hatanaka, Gen Murakami, Hideo Mukai, Yasushi Hojo, Minoru Saito, Tetsuya Kimoto, Suguru Kawato

**Affiliations:** 1 Department of Biophysics and Life Sciences, Graduate School of Arts and Sciences, The University of Tokyo, Tokyo, Japan; 2 Department of Physics, College of Science and Technology, Nihon University, Tokyo, Japan; 3 Bioinformatics Project of Japan Science and Technology Agency, The University of Tokyo, Tokyo, Japan; 4 Department of Correlative Study in Physics and Chemistry, Graduate School of Integrated Basic Sciences, Nihon University, Tokyo, Japan; Max-Delbrück Center for Molecular Medicine (MDC), Germany

## Abstract

**Background:**

Modulation of dendritic spines under acute stress is attracting much attention. Exposure to acute stress induces corticosterone (CORT) secretion from the adrenal cortex, resulting in rapid increase of CORT levels in plasma and the hippocampus.

**Methodology/Principal Findings:**

Here we demonstrated the mechanisms of rapid effect (∼1 h) of CORT on the density and morphology of spines by imaging neurons in adult male rat hippocampal slices. The application of CORT at 100–1000 nM induced a rapid increase in the density of spines of CA1 pyramidal neurons. The density of small-head spines (0.2–0.4 µm) was increased even at low CORT levels (100–200 nM). The density of middle-head spines (0.4–0.5 µm) was increased at high CORT levels between 400–1000 nM. The density of large-head spines (0.5–1.0 µm) was increased only at 1000 nM CORT. Co-administration of RU486, an antagonist of glucocorticoid receptor (GR), abolished the effect of CORT. Blocking a single kinase, such as MAPK, PKA, PKC or PI3K, suppressed CORT-induced enhancement of spinogenesis. Blocking NMDA receptors suppressed the CORT effect.

**Conclusions/Significance:**

These results imply that stress levels of CORT (100–1000 nM) drive the spinogenesis via synaptic GR and multiple kinase pathways.

## Introduction

Stress modulates the functions and architectures of mammalian brain. The influences of stress are elicited at least in part by corticosterone (CORT), which is produced in the adrenal cortex in response to stress. CORT receptors are abundantly expressed in the hippocampus [1] and consequently the hippocampus is sensitive to CORT [2]–[4]. Elevated CORT resulting from chronic stress slowly produces neuronal cell damage in the hippocampus. Rats exposed to restraint stress for 3 weeks exhibit neuronal atrophy and decrease dendritic branches [3]. Exogenous application of a high dose of CORT for 3 weeks (mimicking the effects of prolonged restraint stress) elicits neuronal atrophy and decreases dendritic branches in the hippocampus [4]. There are only a few reports regarding chronic effect of CORT on dendritic spines. As a few examples, 3 weeks administration of CORT induced a decrease in spine density in CA1 hippocampal neurons [5]. These slow steroid effects (occurring over several days) are mediated by nuclear receptors. Upon binding of steroids to nuclear glucocorticoid receptor (GR), GR forms dimer and binds to the glucocorticoid response element of genes, resulting in protein synthesis.

The neuronal response to acute stress (a stressor lasting for a few hours) may be very different from that of chronic stress [6]. CORT rapidly modulates (within 2 h) neuronal activity, which may occur independently of the regulation of the gene expression [7], [8]. For example, stress levels (0.5–10 µM) of CORT rapidly suppress the long-term potentiation (LTP) induced by primed burst stimulation [9] or tetanic stimulation [10]. Furthermore, application of CORT (1–10 µM) rapidly suppresses the NMDA-induced Ca^2+^ elevation in the CA1 region of adult hippocampal slices [11]. On the other hand, little is known about the effects of acute stress on hippocampal spines (postsynaptic structures). As one of a few examples, the administration of corticotropin releasing hormone (CRH), rapidly induces loss of dendritic spines (within 0.5 h) in Yellow Fluorescence Protein (YFP)-expressing hippocampal CA3 neurons [12]. However, the rapid effects of CORT on spinogenesis have not been well elucidated in the hippocampus. Therefore, in the current study we investigate the rapid effects of stressful concentration of CORT on spine density and spine morphology in the hippocampal CA1 region, with particular interest on signaling via synaptic GR and multiple kinase pathways.

## Results

### Corticosterone rapidly promotes spinogenesis

**Figure 1:**
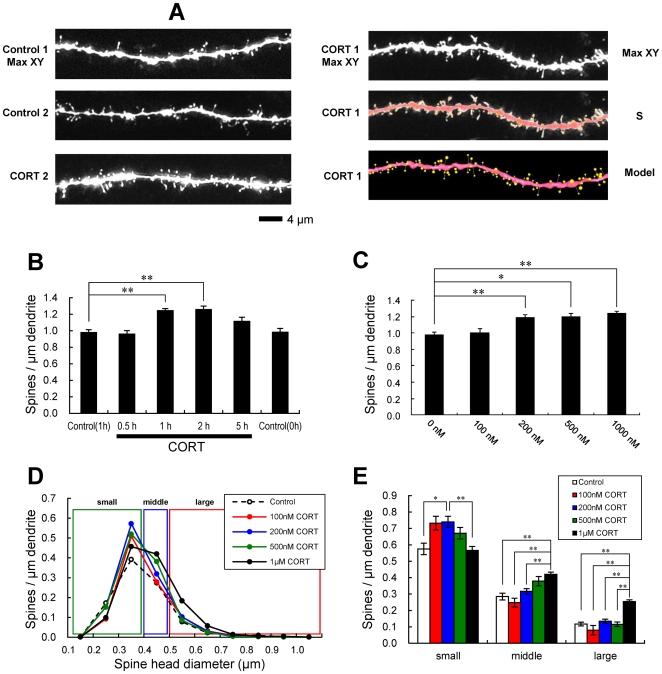
Changes in the density and morphology of spines by CORT in hippocampal slices. Spines were analyzed along the secondary dendrites of pyramidal neurons in the stratum radiatum of CA1 neurons. (A) Maximal intensity projections onto XY plane from z-series confocal micrographs (Max XY), showing spines along the secondary dendrites of hippocampal CA1 pyramidal neurons. Dendrites without drug-treatments (Control 1, 2) and with 1000 nM (1 µM) CORT treatment for 1 h (CORT 1, 2). For CORT 1, spine images analyzed by Spiso-3D (S) and 3 dimensional model illustration (Model) are also shown. Bar, 4 µm. (B) The time dependency of CORT effects on the total spine density in CA1 neurons, after 0.5 h treatment (0.5 h) , 1 h treatment (1 h) , 2 h treatment (2 h), 5 h treatment (5 h) in ACSF with 1 µM CORT. As controls, both 1 h treatment (Control, 1 h) and 0 h treatment (Control, 0 h) in ACSF without CORT are shown. These two controls have identical total spine density. (C) Dose dependency of CORT treatments on the total spine density. A 1 h treatment in ACSF without CORT (0 nM), with 100 nM CORT (100 nM), with 200 nM CORT (200 nM), with 500 nM CORT (500 nM), with 1 µM CORT (1 µM). Vertical axis is the average number of spines per 1 µm of dendrite. (D, E) Dose dependency of CORT effects upon 1 h treatment on spine head diameters. From left to right, small-head spines (small), middle-head spines (middle), and large-head spines (large). A 1 h treatment in ACSF without drugs (Control), with 100 nM CORT (100 nM), with 200 nM CORT (200 nM), with 500 nM CORT (500 nM), with 1 µM CORT (1 µM). Dose dependency showed that the enhancing effect on small-head spines already appears at 100 nM CORT, and that the spine head is enlarged depending on the increase in CORT level. In (B)–(E), vertical axis is the average number of spines per 1 µm of dendrite. In (B), (C) and (E), results are reported as mean ± SEM. In (B), (C) and (E), the significance of CORT or drug effect was examined using the Tukey–Kramer *post hoc* multiple comparisons test when one way ANOVA tests yielded *P*<0.05. *P<0.05, **P<0.01. For each drug treatment, we investigated 3 rats, 6 slices, 12 neurons, 24 dendrites and 1100–1800 spines, except for 1 µM CORT which consists of 10 rats, 28 slices, 56 neurons, 113 dendrites and approx. 8000 spines. For control, we used 5 rats, 8 slices, 16 neurons, 31 dendrites and approx. 1700 spines.

We investigated the effect of stressful concentration of CORT on the modulation of the dendritic spine density and morphology in the hippocampus. To this end, single spine imaging was performed for Lucifer Yellow-injected neurons in hippocampal ‘acute’ slices from 12 week-old male rats (Fig. 1A). We analyzed secondary branches of the apical dendrites located 100–250 µm distant from the pyramidal cell body around the middle of the stratum radiatum of CA1 region (Fig. 1A).

#### Total spine density analysis

Following a 1 h treatment with 1000 nM (1 µM) CORT, treated dendrites had significantly more spines (1.24 spines/µm) than control dendrites (0.98 spines/µm, with no CORT) (Figs. 1A, 1B). However, no significant increase in the density was observed after 0.5 h incubation with 1 µM CORT, indicating that at least 1 h incubation is necessary to change the total spine density. After exposure to 1 µM CORT for 0.5 h, 1 h, 2 h, and 5 h, the total spine density was changed to 0.96 spines/µm, 1.24 spines/µm, 1.26 spines/µm, and 1.11 spines/µm, respectively (Fig. 1B). The density of spines increased at 1–2 h and then decreased at 5 h. Dose dependency was examined after 1 h incubation. Dose dependency showed that the enhancing effect was significant at 200 nM, 500 nM or 1 µM CORT (Fig. 1C). It should be noted that, when we used the spine head diameter analysis, a significant effect was already observed at 100 nM CORT (Figs. 1D, 1E).

**Figure 2:**
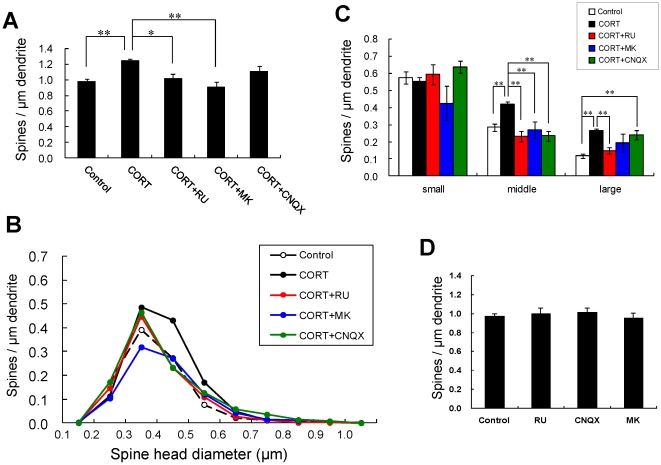
Effects of blockers of receptors on changes by CORT in the density and morphology of spines. (A) Effects of treatments by 1 µM CORT and blockers of receptors on the total spine density in CA1 neurons. A 1 h treatment in ACSF without drugs (Control), with 1 µM CORT (CORT), with 1 µM and 10 µM RU486 (CORT + RU), with 1 µM CORT and 50 µM MK-801 (CORT + MK), with 1 µM CORT and 20 µM CNQX (CORT + CNQX). (B) Histogram of spine head diameters after a 1 h treatment in ACSF without drugs (Control, open circle), with 1 µM CORT (CORT, closed black circle), and with 1 µM CORT and 10 µM RU486 (CORT + RU, closed red circle), with 1 µM CORT and 50 µM MK-801 (CORT + MK, closed blue circle), with 1 µM CORT and 20 µM CNQX (CORT + CNQX, closed green circle). Small-head spines (small), middle-head spines (middle), and large-head spines (large) are categorized. (C) Density of three subtypes of spines. Abbreviations are same as in (B). ACSF without drugs (open column), CORT (black column), CORT + RU (red column), CORT + MK (blue column), and CORT + CNQX (green column) are shown. (D) No effect of receptor inhibitors alone on the total spine density in CA1 neurons. Abbreviations are the same as in (A). Vertical axis is the average number of spines per 1 µm of dendrite. In (A) and (C), results are reported as mean ± SEM. In (A) and (C), the significance of CORT or drug effect was examined using the Tukey–Kramer *post hoc* multiple comparisons test when one way ANOVA tests yielded *P*<0.05. *P<0.05, **P<0.01. For each drug treatment, we investigated 3 rats, 7 slices, 14 neurons, 28 dendrites and 1400–2000 spines, except for CORT which consists of 10 rats, 28 slices, 56 neurons, 113 dendrites and approx. 8000 spines. For control, we used 5 rats, 8 slices, 16 neurons, 31 dendrites and approx. 1700 spines.

A 1 h treatment with 1 µM CORT was used in the kinase inhibitor investigations unless specified, because 1 µM CORT showed the strongest effects. Blocking of GR by 10 µM RU486 completely abolished the enhancing effect by 1 µM CORT on the total spine density (1.01 spines/µm) (Fig. 2A). It should be noted that rapid CORT effects (within 1 h) did not induce neurodegeneration, as judged from no change in dendrites and cell body images (data not shown).

#### Spine head diameter analysis

In order to increase the accuracy of analysis, the morphological changes in spine head diameter were assessed (Figs. 1D, 1E, 2B, 2C). The majority of spines (>95%) had a distinct head and neck, therefore we statistically analyzed these spines having a distinct head. We classified the spines into three categories depending on their head diameters, i.e. small-head spines (0.2–0.4 µm), middle-head spines (0.4–0.5 µm), and large-head spines (0.5–1.1 µm) [13]. The categorization of three subclasses enabled to distinguish different responses in spine subpopulations upon CORT or kinase inhibitor application. Small-, middle-, and large-head spines may have different physiological functions, because the density of 2-amino-3-(5-methyl-3-oxo-1,2- oxazol-4-yl)propanoic acid (AMPA) receptor has a positive correlation with the area of postsynaptic density (PSD) [14]. Conversely, the density of NMDA receptors is inversely correlated with the PSD area.

We performed a statistical analysis based on classification of the spines into above these categories (Figs. 1E, 2C). In control slices (0 nM CORT), the spine density was 0.58 spines/µm for small-head spines, 0.28 spines/µm for middle-head spines, and 0.12 spines/µm for large-head spines. Dose dependency of the density of small-head spines showed a significant increase already at 100 and 200 nM CORT after 1 h treatment. The middle-head spines increased in proportion to the concentration of CORT over 200–1000 nM. The density of large-head spines increased only by 1 µM CORT. In other words, CORT enlarged the spine head in dose dependent manner.

Upon treatment with 1 µM CORT, the density of middle-head and large-head spines increased significantly from 0.28 to 0.42 spines/µm and from 0.12 to 0.25 spines/µm, respectively, while the density of small-head spines was not significantly altered (Fig. 1E). The simultaneous application of CORT and RU486 significantly decreased the densities of middle-head and large-head spines to 0.25 spines/µm and to 0.15 spines/µm, respectively (Fig. 2C).

### CORT enhances spinogenesis via MAPK, PKA, and PKC pathways

**Figure 3:**
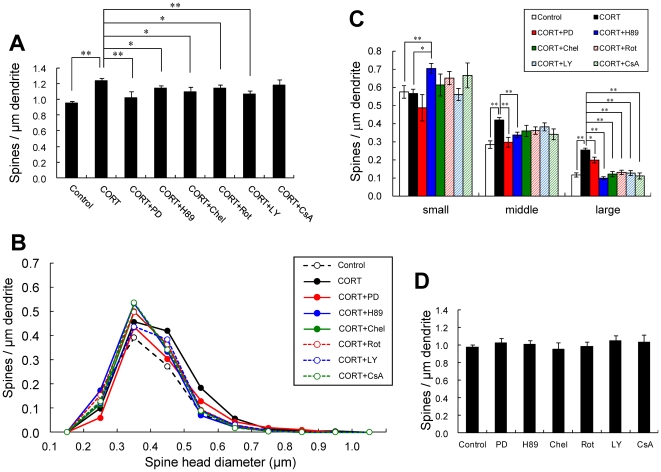
Suppression effects by kinase inhibitors on CORT-induced changes in the density and morphology of spines. (A) Total spine density. Effect of kinase inhibitors in the presence of CORT in CA1 neurons. A 1 h treatment in ACSF without drugs (Control), with 1 µM CORT (CORT), with 1 µM CORT and 20 µM PD98059 (MAPK inhibitor) (CORT + PD), with 1 µM CORT and 10 µM H-89 (PKA inhibitor) (CORT + H89), with 1 µM CORT and 10 µM chelerythrine (PKC inhibitor) (CORT + Chel), with 1 µM CORT and 10 µM rottlerin (PKC delta inhibitor) (CORT + Rot), with 1 µM CORT and 10 µM LY294002 (PI3K inhibitor) (CORT + LY), and with 1 µM CORT and 1 µM cyclosporin A (calcineurin inhibitor) (CORT + CsA). Concentrations of inhibitors are moderate levels. (B) Histogram of spine head diameters. Abbreviations are the same as in (A). After a 1 h treatment in ACSF without drugs (Control, open circle), with CORT (closed black circle), with CORT + PD (closed red circle), with CORT + H89 (closed blue circle), with CORT + Chel (closed green circle), with CORT + Rot (open red circle), with CORT + LY (open blue circle) and with CORT + CsA (open green circle). (C) Density of three subtypes of spines. Abbreviations are the same as in (A). From left to right, small-head spines (small), middle-head spines (middle), and large-head spines (large). In each group, Control (open column), CORT (black column), CORT + PD (red column), CORT + H89 (blue column), CORT + Chel (green column), CORT + Rot (hatched red column), CORT + LY (hatched blue column), and CORT + CsA (hatched green column). (D) No effect of kinase inhibitors alone on the total spine density in CA1 neurons. Abbreviations are the same as in (A). Vertical axis is the average number of spines per 1 µm of dendrite. In (A), (C) and (D), results are reported as mean ± SEM. In (A) and (C), the significance of CORT or drug effect was examined using the Tukey–Kramer *post hoc* multiple comparisons test when one way ANOVA tests yielded *P*<0.05. *P<0.05, **P<0.01. For each drug treatment, we investigated 3 rats, 7 slices, 14 neurons, 28 dendrites and 1400–2000 spines, except for 1 µM CORT which consists of 10 rats, 28 slices, 56 neurons, 113 dendrites and approx. 8000 spines. For control, we used 5 rats, 8 slices, 16 neurons, 31 dendrites and approx. 1700 spines.

Next we characterized intracellular signaling pathways involved in spinogenesis induced by CORT. We investigated the contribution of several essential kinases to the spine density using specific inhibitors (Fig. 3).

#### Total spine density analysis

Blocking of Erk MAPK by application of 20 µM PD98059 (PD) [15], abolished the CORT effect on the increase of spine density of CORT. Application of 10 µM H89, a protein A kinase inhibitor [16], prevented the effect by CORT. Application of 10 µM chelerythrine (Chel), an inhibitor of all the PKC species (alpha, delta, and epsilon) [17], partially prevented the effect by CORT. Application of 10 µM rottlerin (Rot), a selective inhibitor of PKC delta [18], prevented the effect by CORT. Application of 10 µM LY294002 (LY), a PI3K inhibitor [19], prevented the effect by CORT. On the other hand, cyclosporin A (CsA), an inhibitor of calcineurin (PP2B) [20], did not alter the effect of CORT (1.18 spines/µm). Because the concentrations of inhibitors added are recommended levels, the observed inhibitory effects are not non-specific, due to excess amount of inhibitors. It should be noted that these kinase inhibitors alone did not significantly affect the total spine density within experimental error, indicating that the observed inhibitory effects are not due to simple blocker's non-specific suppressive effects (Fig. 3D).

#### Spine head diameter analysis

Because the total spine density was not sensitive enough to describe complex kinase effects, the changes of spine head diameter distribution were analyzed (Figs. 3B, 3C). For example, even though the total spine density was not very much altered by H89 and CsA as compared with the spine populations in the presence of only CORT, H89 and CsA decreased the density of large-head spines but increased the density of small-head spines.

Blocking Erk MAPK (PD) abolished the effect of the spine density of CORT, decreasing the density of large-head spines from 0.26 to 0.20 spines/µm and middle-head spines from 0.43 to 0.30 spines/µm, while significant change was not observed in small-head spines (from 0.55 to 0.49 spines/µm). Inhibiting PKA (H89) decreased the density of large-head spines to 0.10 spines/µm and middle-head spines to 0.34 spines/µm, but with a great increase in small-head spines to 0.70 spines/µm. Inhibiting PKC (Chel) also decreased the density of large-head spines to 0.12 spines/µm and middle-head spines to 0.36 spines/µm, but with an increase in small-head spines 0.61 spines/µm. Inhibiting PKCδ (Rot) decreased the density of large-head spines to 0.13 spines/µm and middle-head spines to 0.36, but with an increase in small-head spines 0.65 spines/µm. Inhibiting PI3K (LY) decreased the density of large-head spines to 0.13 spines/µm, but with an increase in small-head spines to 0.70 spines/µm. LY was partially effective in decreasing the density of middle-head spines (0.38 spines/µm). Although CsA did not significantly change the total spine density, CsA decreased the density of large-head spines and middle-head spines, but increased small-head spines. All the kinase inhibitors tested decreased the density of large-head spines significantly from the level in the presence of only CORT (Fig. 3C). Because the concentrations of inhibitors added are moderate levels, the observed inhibitory effect cannot be non-specific, due to excess amount of inhibitors. It should be noted that these only kinase inhibitors did not significantly affect the distribution of spine head diameter within experimental error, indicating that the observed inhibitory effects are not due to simple blocker's poison effects (Figs. 3D, S2, S3).

### Blocking of glutamate receptors abolished CORT-induced spinogenesis

We investigated the importance of Ca^2+^ homeostasis within spines on CORT effects. Because the Ca^2+^ level may be maintained with spontaneous fluctuation of opening/closing via ionotropic glutamate receptors in spines, we examined spinogenesis in the presence of inhibitors of these receptors.

#### Total spine density

MK-801, an NMDA receptor blocker, abolished the CORT effect resulting in a significant decrease in the total spine density to 0.90 spines/µm. 6-cyano-7-nitroquinoxaline-2,3-dione (CNQX), an inhibitor of AMPA receptor, only weakly suppressed the effect of CORT on the spine density to 1.07 spines/µm (Fig. 2A).

#### Spine head diameter analysis

Inhibition of NMDA receptors abolished the effect of CORT by significantly decreasing the density of middle-head spines, with a weak decrease in large-head and small-head spines. CNQX decreased middle spines, but did not significantly change the large- and small-head spines. In addition CORT-treatment with Ca^2+^ free ACSF did not induce the increase of spines. These data suggest that the basal Ca^2+^ level kept via spontaneous Ca^2+^ influx/efflux through NMDA receptors is necessary for CORT-induced spinogenesis.

### CORT-induced spinogenesis with protein synthesis

**Figure 4:**
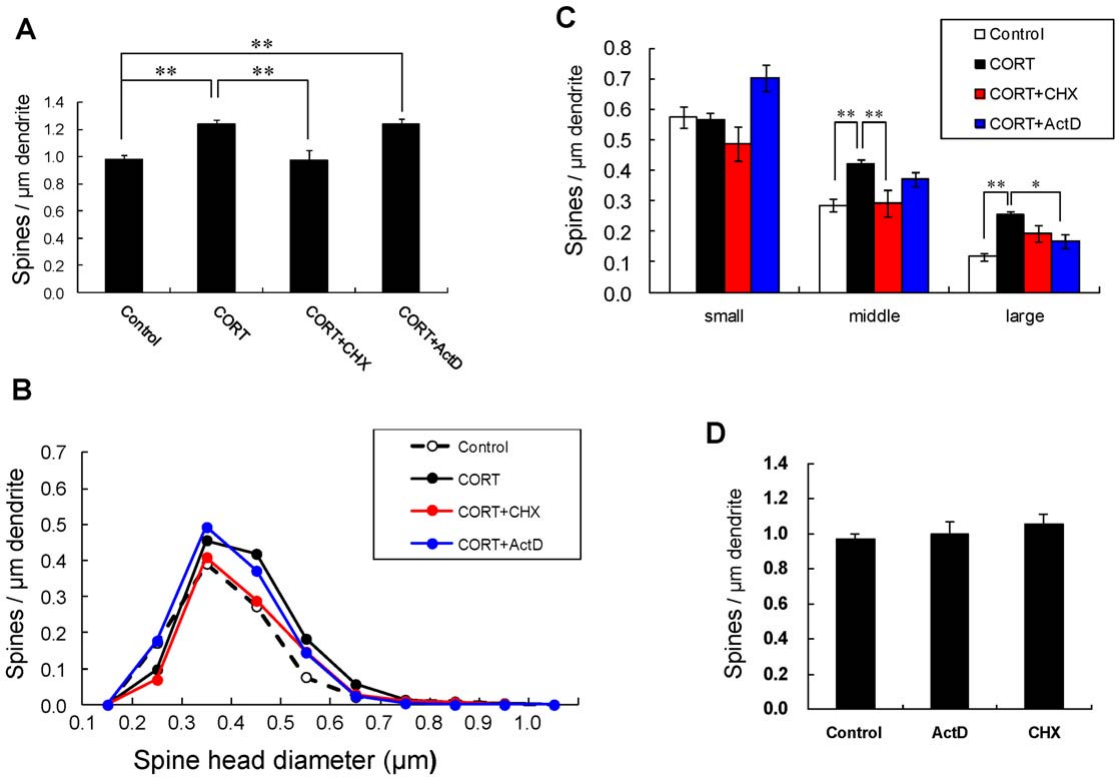
Effects of inhibitors for protein and mRNA synthesis on changes in the density and morphology of spines by CORT. (A) Total spine density. Effect of inhibitors for protein or mRNA synthesis in the presence of CORT on CA1 neurons. A 1 h treatment in ACSF without drugs (Control), with 1 µM CORT (CORT), with 1 µM CORT and 20 µM cycloheximide (CORT + CHX), and with 1 µM CORT and 4 µM actinomycin D (CORT + ActD). (B) Histogram of spine head diameters. Abbreviations are same as in (A). Control (open circle), CORT (closed black circle), CORT + CHX (closed red circle), and CORT + ActD (closed blue circle). (C) Density of three subtypes of spines. Abbreviations are same as in (A). From left to right, small-head spines (small), middle-head spines (middle), and large-head spines (large). ACSF without drugs (open column), CORT (black column), CORT + CHX (red column), and CORT + ActD (blue column). Vertical axis is the average number of spines per 1 µm of dendrite. Statistical significance is calculated against CORT treated group in each spine subtypes and comparisons reached significance are indicated by stars. (D) No effect of inhibitors alone for protein and mRNA synthesis on the total spine density in CA1 neurons. Abbreviations are the same as in (A). Vertical axis is the average number of spines per 1 µm of dendrite. In (A) and (C), results are reported as mean ± SEM. In (A) and (C), the significance of CORT or drug effect was examined using the Tukey–Kramer *post hoc* multiple comparisons test when one way ANOVA tests yielded *P*<0.05. *P<0.05, **P<0.01. For each drug treatment, we investigated 3 rats, 6 slices, 12 neurons, 24 dendrites and 1100–1800 spines, except for 1 µM CORT which consists of 10 rats, 28 slices, 56 neurons, 113 dendrites and approx. 8000 spines. For control, we used 5 rats, 8 slices, 16 neurons, 31 dendrites and approx. 1700 spines.

Cycloheximide (CHX), a protein synthesis inhibitor, altered the effect of CORT-induced increase in the density of spines. CHX decreased the total density to 0.97 spines/µm, by decreasing the density of middle-head spines (Fig. 4).

Actinomycin D (ActD), an mRNA synthesis inhibitor, did not suppress the CORT-induced increase in the total density of spines (Fig. 4). Spine head diameter analysis showed that ActD reduced large-head spines, but increased small-head spines, resulting in restoration of the total spine density to the same level as that with only CORT.

### Spine density in hippocampal slices from ADX rats

**Figure 5:**
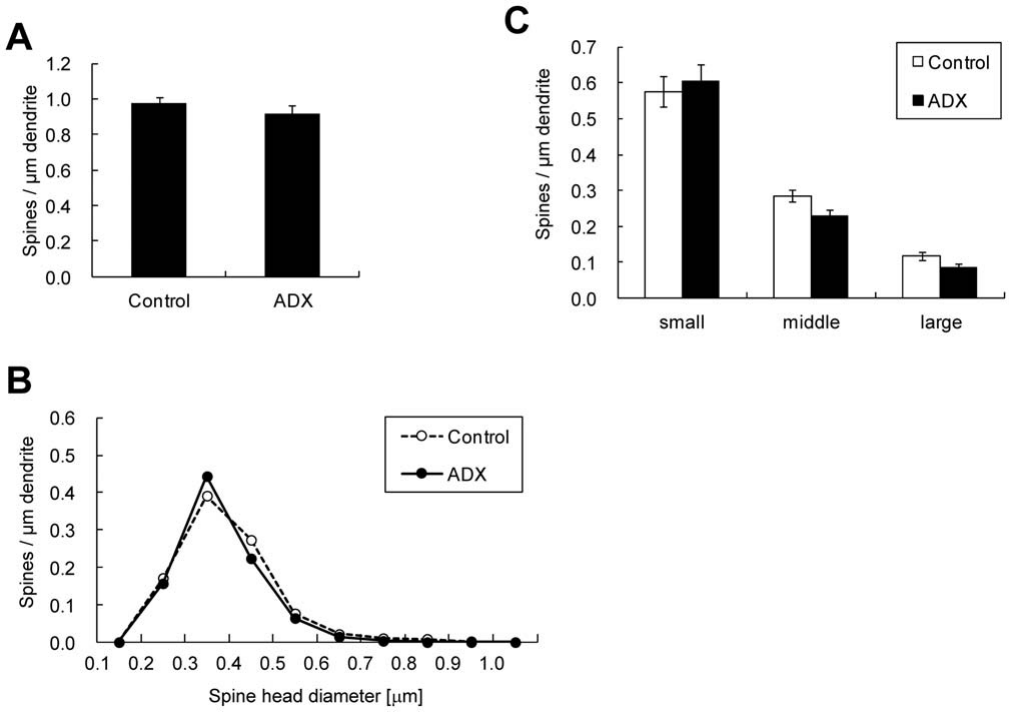
Spine density of hippocampal CA1 neurons in sham rat (Control) or adrenalectomized rat (ADX). (A) Total spine density. For both sham and ADX rats, slices are incubated for 1 h in ACSF without drugs. The Total spine density was not different significantly between Control and ADX rats. (B) Histogram of spine head diameters. Abbreviations are same as in (A). After a 1 h treatment in ACSF without drugs for sham rat (Control, open circle) and ADX (closed black circle). (C) Density of three subtypes of spines. From left to right, small-head spines (small), middle-head spines (middle), and large-head spines (large). Abbreviations are same as in (A). Control (open column) and ADX (black column). Vertical axis is the average number of spines per 1 µm of dendrite. In (A) and (C) results are reported as mean ± SEM. The significance was examined using the Tukey–Kramer *post hoc* multiple comparisons test when one way ANOVA tests yielded *P*<0.05. For sham and ADX rats, we investigated 3 rats, 6 slices, 12 neurons, 24 dendrites and approx. 1300 spines.

To analyze the spines under the CORT depleted conditions *in vivo* for 1 week, we measured the spine density in hippocampal slices from ADX rats. ‘Acute’ hippocampal slices were prepared from sham and ADX rats as described in Materials and Methods. The total spine density of CA1 neurons was not significantly different between control sham rats (0.98±0.04 spines/µm) and ADX rats (0.92±0.05 spines/µm). Spine morphology (head diameter distribution) was also not significantly different between control rats and ADX rats (Fig. 5).

### Time-dependent morphological changes of spines by 1 µM CORT

The morphological change of spines was investigated between 1 and 5 h (Fig. S1). Following 1 h, 2 h and 5 h treatments with 1 µM CORT, the density of large-head spines increased at 1 h and then decreased back to the control level at 2 and 5 h. The density of middle-head spines increased at 1 and 2 h and then decreased back to the control level at 5 h. Small-head spines did not increase at 1 h but increased significantly at 2 and 5 h.

### Mass-spectrometric analysis of CORT in slices

The concentration of CORT was determined by mass-spectrometric analysis [21]. In hippocampal ‘acute’ slices used for spine analysis, after recovery incubation in steroid-free ACSF for 2 h, the level of CORT was 0.67±0.07 ng/g wet weight (i.e., 1.9±0.2 nM, n = 5). Before recovery incubation in ACSF, hippocampal CORT was 128±39 ng/mL (i.e., 370±111 nM, n = 8), due to ether stress before decapitation. The level of plasma CORT was 510±58 ng/mL (i.e., 1473±168 nM, n = 8) due to ether stress before decapitation. On the other hand, the level of plasma CORT was only 0.8 ng/mL (i.e., 2.3 nM) in ADX rats, even after ether stress and decapitation. For additional descriptions see Text S1.

## Discussion

We demonstrated GR- and kinase-dependent mechanisms of rapid CORT-induced spinogenesis.

### Contribution of synaptic/extranuclear GR to the rapid modulation

The current study showed that 100–1000 nM CORT enhanced spinogenesis in CA1 hippocampal neurons mediated by GR. Treatment with CORT for 1 h resulted in a significant increase of the spine density and enlargement of the spine head (toward middle-head or large-head).

The current CORT treatment may rapidly activate the synaptic GR. We observed GR localized within the postsynaptic structures via postembedding immunogold staining (Supporting Information, Results and Fig. S4). GR was often located in the cytoplasm of spines and in some cases at PSD area [22] (Text S1 and Fig. S4). GR was observed also in dendrites [22]. We identified GR expression also in purified PSD fraction by Western blot analysis [22], [23]. These results suggest that the rapid modulation of spines by CORT is mediated by postsynaptic or extranuclear GR. The involvement of GR in the CORT effect was also supported by GR antagonist RU486 which blocked CORT-induced spinogenesis (Fig. 2). In addition, GR agonist dexamethasone induces rapid spinogenesis in hippocampal CA1 neurons within 1 h [23]. Membrane GR-induced rapid PKA activation (∼1 h) has been demonstrated for inhibitory avoidance behavior via rat basolateral amygdala [24], suggesting that synaptic GR may activate kinases.

Since in our ‘acute’ slice CORT level is roughly 2 nM, all MR may be occupied due to high affinity (Kd ∼0.5 nM) to aldosterone or CORT. Therefore the effect of CORT on spines is mainly mediated by GR but not by MR, in the presence of 1 µM CORT. The 1 h responses may be too rapid for nuclear GR actions which often need more than 5 h due to genetic processes. As an another possibility, these rapid actions may include rapid genomic actions via nuclear GR, which are suggested as a reduction of dendritic length (1–4 h) [25] or an impairment of enhancement of voltage-dependent Ca^2+^ currents in mutated GR (1–4 h) [26], in hippocampal neurons. However, a significant suppression of spine changes by application of kinase inhibitors (Fig. 3) may put more weight on GR–kinase pathway rather than DNA binding of GR.

Since RU486 suppresses not only GR but also progesterone receptor (PR) in Fig. 2, progesterone (PROG) effect should be considered. The treatment of slices with 10 nM PROG for 1 h did not significantly increase the spine density within experimental error (data not shown), excluding the involvement of PROG and PR in the observed spinogenesis.

### CORT enhances spinogenesis via kinase networks and their downstream (model illustration in Fig. S5)

Classification of individual spine head diameters using the criteria of small-, middle-, and large-head (three classes), is particularly useful to distinguish different effects of many kinases on the CORT-induced spinogenesis as compared with previous classification of mushroom or thin (only two classes) (Fig. 1E). The classification of spines in three classes based on spine diameter is also used successfully in previous studies [27]. Note that large-head spines may have higher memory capacity than middle- and small-head spines, because large-head spines have more AMPA receptors than middle- and small-head spines [14]. The density of NMDA receptor is roughly independent of the size of spine head.

CORT rapidly increased not only the spine density but also the spine head diameter (Fig. 3). Since the density of large-head spines in the presence of 1 µM CORT were significantly decreased upon inhibition of Erk MAPK, PKA, PKC or PI3K, these kinases probably participate in the enlargement of spine heads. At the moment, we cannot distinguish between two possible mechanisms about enlargement of spines (e.g., either CORT increased small-head spines then enlarged them to larger head spines or CORT newly induced spines with larger sizes).

In earlier studies, rapid MAPK activation (∼2 h) via GR has been demonstrated in the mice hippocampus or pituitary-derived cell-lines AtT20 [28]. The expression of Raf1, Ras, p-MAPK is elevated rapidly upon application of 10 nM CORT. Fear conditioning of mice is dependent on GR–MAPK pathway. Rapid PKA activation (phosphorylation of PKA) (∼1 h) via membrane located GR has been demonstrated in rat basolateral amygdala [24].

In CA1 region, MAPK cascade is known to couple with PKA and PKC via PKC→Raf1→MAPK, PKA→B-Raf→MAPK in synaptic modulation including LTP (Roberson et al., 1999). Take the knowledge into account, MAPK may be a key kinase responsible for modulation of spines.

What is the target of Erk MAPK in spine reorganization? Erk MAPK is known to phosphorylate cortactin, a structural protein associated to actin [29]. Cortactin interacts with both F-actin and actin-related protein (Arp) 2/3 complex as well as scaffold protein Shank in the PSD at the SH3 domain [30], [31], resulting in promotion of actin fiber remodeling within spines.

It is thus probable that CORT exerts its effect on spines via cortactin-actin pathway. Cortactin has multiple phosphorylation sites which are activated by MAPK [32]. Phosphorylation of cortaction may promote assembly of actin cytoskeletal matrices, resulting in spine formation or modulation of spine morphology [33]. These sites are putative phosphorylation sites also for other serine/threonine kinase (PKA or PKC) that are activated by CORT. PI3K may also phosphorylate multiple phosphorylation sites of cortactin, resulting in actin polymerization [32].

In the case of *in vivo* hippocampus, the similar rapid CORT-induced modulation of spines might occur. In response to acute severe stress (ether stress), elevation of plasma CORT (to 1–2 µM) occurs, resulting in elevation of hippocampal CORT to 0.5–1 µM, due to penetration of CORT into hippocampus after crossing the Blood Brain Barrier [21]. This increase of CORT level should affect spinogenesis *in vivo*.

### Earlier studies examining rapid CORT effects

There is increasing evidence implying that CORT is capable of driving rapid signaling (around 1 h), independent of slow transcriptional signaling.

High dose of dexamethasone (DEX, GR agonist) injection rapidly (<2 h) activates PI3K via GR in cerebral vascular endothelial cell, and this event is blocked by RU486 [34]. DEX rapidly acts to inhibit recruitment of MAPK to activate EGF receptors in adenocarcinoma cell [35]. Stress levels (approximately 0.6–2.7 µM) of CORT rapidly suppress the long-term potentiation (LTP) *in vivo* induced by primed burst stimulation [9]. However, analysis of possible participation of many other kinases in these rapid CORT signaling are not performed.

### Steroid levels in ‘acute’ slices used for spine experiments

Following exposure to stress in rat, a high-dose of CORT (about 1 µM) is secreted by the adrenal cortex and readily penetrates into the brain from the blood circulation. The steroid levels in slices used for spine analysis must be known. From the current study, the CORT concentration in the freshly isolated hippocampus was 400–1000 nM as determined by mass-spectrometric analysis, because rats were under ether stress for 1 min before decapitation, resulting in penetration of elevated plasma CORT (1–2 µM) into the hippocampus (see Fig. S6) [36]. Note that 1–2 µM CORT is more than the upper limit capacity (400–600 nM) of CORT binding globulin [37]. In the ‘acute’ slices used for our spine analysis, this high concentration of CORT (400–1000 nM) is decreased to a very low level of approx. 2 nM by incubating ‘fresh’ slices in steroid-free ACSF for 2 h, due to leakage of CORT from slices to ACSF [22]. Therefore, we found the enhanced spinogenesis upon increase of CORT level from approx. 2 nM (control) to 100–1000 nM CORT by CORT application. On the other hand, we observed that ADX rats had the low hippocampal CORT of approx. 7 nM in fresh hippocampal slices (even after exposure to ether stress before decapitation) [21]. The slice CORT level further decreased to 2 nM in ‘acute’ slices due to CORT depletion by ACSF incubation. Note that in ADX rats without the adrenal glands, ether stress did not elevate the circulating CORT more than approx. 2.3 nM [21]. Therefore, the CORT level in ‘acute’ slices used for spine experiments was almost identical between adrenal-intact and ADX rats. This may be the reason why the spine density and morphology were almost identical between slices from adrenal-intact rats and slices from ADX rats.

The hippocampus endogenously synthesizes CORT (∼7 nM) [21], therefore, hippocampal CORT may be sufficient to maintain basal spine structures, even in ADX rats. In adrenal intact rats, CORT in circulation may be ∼20 nM [38], [39], thereby the hippocampal CORT level might not be considerably different between adrenal intact and ADX rats, by considering CORT penetrated from plasma and the hippocampus-synthesized CORT.

### Other examples of kinase-dependent spinogenesis

The activation of synaptic androgen receptor AR by testosterone or dihydrotestosterone induces a rapid increase of thorns of thorny excrescences in CA3 pyramidal neurons of adult rat hippocampal ‘acute’ slices within 2 h. Here, thorns are spine-like postsynaptic structures of CA3 pyramidal neurons. The rapid synaptic action of androgen is mediated by activation of many kinases. For example, the thorn-genesis induced by androgen is mediated by Erk MAPK, p38 MAPK, PKC, CaMKII, and calcineurin, but not by PKA, PI3K, and PKCδ [40]. The rapid spinogenesis by estradiol in CA1 and CA3 pyramidal neurons of hippocampal ‘acute’ slices is mediated by synaptic ERα→Erk MAPK pathway [41], [42].

### Conclusion

Upon CORT application at 100–1000 nM for 1 h, in order to mimic the acute stress, the rapid increase occurred in the density of small-, middle- and large-head spines, significantly depending on the concentration of CORT. This modulation by CORT was mediated by synaptic GR and multiple kinase networks including PKA, PKA and MAPK. Very different from chronic stress, which impairs neurons (e.g., neuronal atrophy, decrease in dendritic branches and decrease in the density of spines), acute stress may activate neurons via increase of the density of spines or enlargement of spine heads.

## Materials and Methods

### Animals

Male Wistar rats were purchased from Saitama Experimental Animal Supply (Japan). All animals were maintained under a 12 h light/12 dark exposure and free access to food and water. Adrenalectomy (ADX) and sham operation were performed one-week before the experiments. The experimental procedure of this research was approved by the Committee for Animal Research of Univ of Tokyo.

### Chemicals

Corticosterone, actinomycin D, cyano-nitroquinoxaline-dione (CNQX), cycloheximide, cyclosporin A, LY294002, MK-801, PD98059, RU486 and Lucifer Yellow CH were purchased from Sigma (USA). Chelerythrine and rottlerin were purchased from Calbiochem (Germany). H-89 was purchased from Biomol (USA).

### Slice preparation

Twelve weeks male rats were deeply anesthetized with ethyl ether and decapitated between 9:00 h and 10:00 h when plasma CORT levels are low. Immediately after decapitation, the brain was removed from the skull and placed in ice-cold oxygenated (95% O_2_, 5% CO_2_) artificial cerebrospinal fluid (ACSF) containing (in mM): 124 NaCl, 5 KCl, 1.25 NaH_2_PO_4_, 2 MgSO_4_, 2 CaCl_2_, 22 NaHCO_3_, and 10 D-glucose (all from Wako); pH was set at 7.4. Hippocampal slices, 400 µm thick, were prepared with a vibratome (Dosaka, Japan). These slices were ‘fresh’ slices without ACSF incubation. Slices were then incubated in oxygenated ACSF for 2 h (slice recovery processes) in order to obtain widely used ‘acute slices’. These ‘acute’ slices were then incubated at room temperature with CORT or other drugs such as kinase inhibitors. Then, slices were prefixed with 4% paraformaldehyde at 4°C for 4 h.

### Imaging and Analysis of Dendritic Spine Density and Morphology

Spine imaging and analysis with confocal microscopy was performed essentially as described previously [23], [41], [43].

#### Current injection of Lucifer Yellow

Neurons within slices were visualized by an injection of Lucifer Yellow under a Nikon E600FN microscope (Nikon, Japan) equipped with a C2400–79H infrared camera (Hamamatsu Photonics, Japan) and with a 40× water immersion lens (Nikon, Japan). Dye was injected with a glass electrode whose tip was filled with 5% Lucifer Yellow for 15 min, using Axopatch 200B (Axon Instruments, USA). Approximately five neurons within a 100–200 µm depth from the surface of a slice were injected [44].

#### Confocal laser microscopy and morphological analysis

The imaging was performed from sequential z-series scans with LSM5 PASCAL confocal microscope (Carl Zeiss, Germany). For analysis of spines, three-dimensional images were constructed from approximately 40 sequential z-series sections of neurons scanned every 0.45 µm with a 63× water immersion lens, NA 1.2 (Carl Zeiss, Germany). For Lucifer Yellow, the excitation and emission wavelengths were 488 nm and 515 nm, respectively. The applied zoom factor (3.0) yielded 23 pixels per 1 µm. The z-axis resolution was approximately 0.71 µm. The confocal lateral resolution was approximately 0.26 µm. The limits of resolution were regarded as sufficient to allow the determination of the density of thorns or spines. Confocal images were then deconvoluted using AutoDeblur software (AutoQuant, USA). The density of spines as well as the head diameter was analyzed with Spiso-3D (automated software mathematically calculating geometrical parameters of spines) developed by Bioinformatics Project of Kawato's group [13]. Results obtained by Spiso-3D is almost identical to those by Neurolucida (MicroBrightField, USA) within assessment difference of 2%, and Spiso-3D considerably reduces human errors and experimental labor of manual software [13]. We analyzed the secondary dendrites in the stratum radiatum, lying between 100 and 250 µm from the soma. The spine density was calculated from the number of spines having a total length of 50–80 µm. Spine shapes were classified into three categories as follows. (1) A small-head spine whose head diameter is between 0.2–0.4 µm. (2) A middle-head spine whose head diameter is between 0.4–0.5 µm. (3) A large-head spine whose head diameter is between 0.5–1.1 µm. These three categories were useful to distinguish different responses upon kinase inhibitor application. Because the majority of spines (>95%) had a distinct head and neck, and stubby spines and filopodium did not contribute much to the overall changes, we analyzed spines having a distinct head.

All protrusions from the dendrites were treated as ‘spines’, although with confocal microscopy, it was not possible to determine whether they formed synapses, or whether some of them were filopodia protrusions which did not form synapses [45]. While counting the spines in the reconstructed images, the position and verification of spines was aided by rotation of three-dimensional reconstructions and by observation of the images in consecutive single planes. As an example, the whole neuron image, with indication of secondary dendrites, stained by Lucifer Yellow is shown in Fig. S7. No significant changes were observed by acute CORT application in dendrite structures.

### Statistical analysis

All the data are expressed as means ± SEM. The significance of CORT or drug effect was examined using the Tukey–Kramer *post hoc* multiple comparisons test when one way ANOVA tests yielded P<0.05.

**Methods for Immunogold microscopy and Mass-spectrometric analysis of CORT** are described in Text S1.

## Supporting Information

Figure S1 Time dependent changes and dose dependent changes in spine morphology by CORT in CA1 neurons. (A, B) The time dependency of CORT effects on spine head diameters. Histogram of spine head diameters, after 1 h treatment (1 h), 2 h treatment (2 h), 5 h treatment in ACSF with 1 µM CORT (5 h). As controls, both 1 h treatment (Control, 1 h) and 0 h treatment (Control, 0 h) in ACSF without CORT are shown. These two controls have identical spine density. Vertical axis is the average number of spines per 1 µm of dendrite. After exposure to CORT for 2 h and 5 h, the small-head spines significantly increased, and the middle- and large-head spines decreased. For each drug treatment, we investigated 3 rats, 6 slices, 12 neurons, 24 dendrites and 1100–1800 spines, except for 1 µM CORT for 1 h which consists of 10 rats, 28 slices, 56 neurons, 113 dendrites and approx. 8000 spines. For control, we used 5 rats, 8 slices, 16 neurons, 31 dendrites and approx. 1700 spines. In (B), the significance of CORT effect was examined using the Tukey–Kramer *post hoc* multiple comparisons test when one way ANOVA tests yielded *P*<0.05. *P<0.05, **P<0.01.
(TIFF)

Figure S2 Images for effects by inhibition of kinases on spinogenesis by CORT. Maximal intensity projections onto XY plane from z-series confocal micrographs, showing spines along the secondary dendrites of hippocampal CA1 pyramidal neurons. Dendrites were treated with 1 µM CORT and 20 µM PD98059 (CORT + PD), with 1 µM CORT and 10 µM H-89 (CORT + H89), with 1 µM CORT and 10 µM chelerythrine (CORT + Chel), with 1 µM CORT and 10 µM rottlerin (CORT + Rot), and with 1 µM CORT and 10 µM LY294002 (CORT + LY) for 1 h. Bar, 4 µm.
(TIFF)

Figure S3 No effect of kinase inhibitors alone on the density of three subtypes of spines in CA1 neurons. A 1 h treatment in ACSF without drugs (Control, open column), with 20 µM PD98059 (PD, red column), with 10 µM H-89 (H89, blue column), with 10 µM chelerythrine (Chel, green column), with 10 µM rottlerin (Rot, hatched red column), with 10 µM LY294002 (LY, hatched blue column), and with 1 µM cyclosporin A (CsA, hatched green column). From left to right, small-head spines (small), middle-head spines (middle), and large-head spines (large). Vertical axis is the average number of spines per 1 µm of dendrite. Statistically significant differences were not observed upon inhibitor alone treatment. Results are reported as mean ± SEM. For each drug treatment, we investigated 3 rats, 6 slices, 12 neurons, 24 dendrites and 1200–1500 spines. The significance of CORT or drug effect was examined using the Tukey–Kramer *post hoc* multiple comparisons test when one way ANOVA tests yielded *P*<0.05.
(TIFF)

Figure S4 Immunoelectron microscopic analysis of the distribution of GR within the axospinous synapses in the stratum radiatum of the hippocampal principal neurons of the CA1 (A)–(D). Gold particles (arrowheads), specifically indicating the presence of GR, were localized in the presynaptic and postsynaptic regions. In dendritic spines, gold particles were found within the spine head and, in some cases were associated with PSD regions, axospinous synapses of the hippocampal principal neurons in the stratum radiatum of the CA1. A search for immunogold-labeled GR proteins was performed for at least 30 synapses at the CA1 region from more than 100 independent images. A 1∶1000 dilution of antiserum was used to prevent nonspecific labeling. Preadsorption of the antibody with GR antigen (30 µg/ml) resulted in the disappearance of immunoreactivity. Pre, presynaptic region; post, postsynaptic region. Scale bar, 200 nm.
(TIF)

Figure S5 Model illustration. (A) Schematic illustration of CORT-driven multiple kinase pathways. Upon binding of CORT, GR induces the sequential activation of PKA, PKC, MEK, and Erk MAPK. Erk MAPK regulates phosphorylation of actin-related proteins such as cortactin, resulting in actin reorganization. (B) Schematic illustration of CORT-induced rapid spinogenesis via multiple kinase pathways.
(TIFF)

Figure S6 Depletion of CORT and other sex-steroids occurs by ACSF incubation to obtain ‘acute’ slices which are used widely for investigations of spinogenesis and electrophysiology.
(TIFF)

Figure S7 A confocal micrograph showing Lucifer Yellow-injected whole neuron morphology including cell body and dendrites of hippocampal CA1 pyramidal neurons. No significant changes were observed in whole neuron shape and dendrite structures before and after CORT application for 1 h. Secondary dendritic spines have been shown to be highly sensitive to sex-steroid hormones from many early studies. Vertical bar 100 µm.
(TIFF)

Text S1 Supporting information for materials and methods, and results.
(DOC)
