# Supplementary figures and images for: Corticosterone Induces Rapid Spinogenesis via Synaptic Glucocorticoid Receptors and Kinase Networks in Hippocampus
Source: PLoS One. 2012 Apr 11;7(4):e34124. doi: 10.1371/journal.pone.0034124 (PMC3324490; doi:10.1371/journal.pone.0034124)

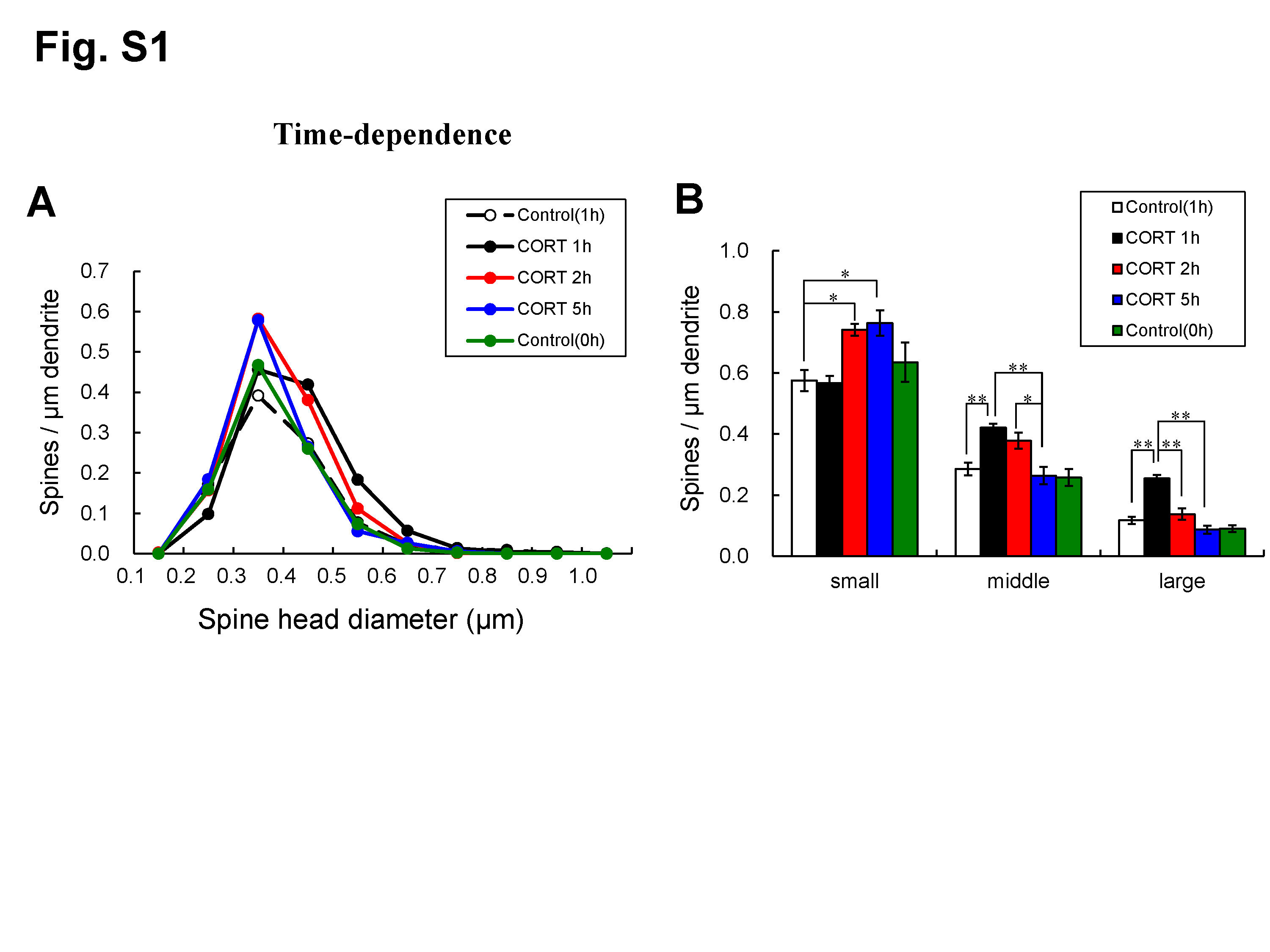

Supplement: Figure S1 — Time dependent changes and dose dependent changes in spine morphology by CORT in CA1 neurons. (A, B) The time dependency of CORT effects on spine head diameters. Histogram of spine head diameters, after 1 h treatment (1 h), 2 h treatment (2 h), 5 h treatment in ACSF with 1 µM CORT (5 h). As controls, both 1 h treatment (Control, 1 h) and 0 h treatment (Control, 0 h) in ACSF without CORT are shown. These two controls have identical spine density. Vertical axis is the average number of spines per 1 µm of dendrite. After exposure to CORT for 2 h and 5 h, the small-head spines significantly increased, and the middle- and large-head spines decreased. For each drug treatment, we investigated 3 rats, 6 slices, 12 neurons, 24 dendrites and 1100–1800 spines, except for 1 µM CORT for 1 h which consists of 10 rats, 28 slices, 56 neurons, 113 dendrites and approx. 8000 spines. For control, we used 5 rats, 8 slices, 16 neurons, 31 dendrites and approx. 1700 spines. In (B), the significance of CORT effect was examined using the Tukey–Kramer post hoc multiple comparisons test when one way ANOVA tests yielded P<0.05. *P<0.05, **P<0.01. (TIFF) [file pone.0034124.s001.tiff]

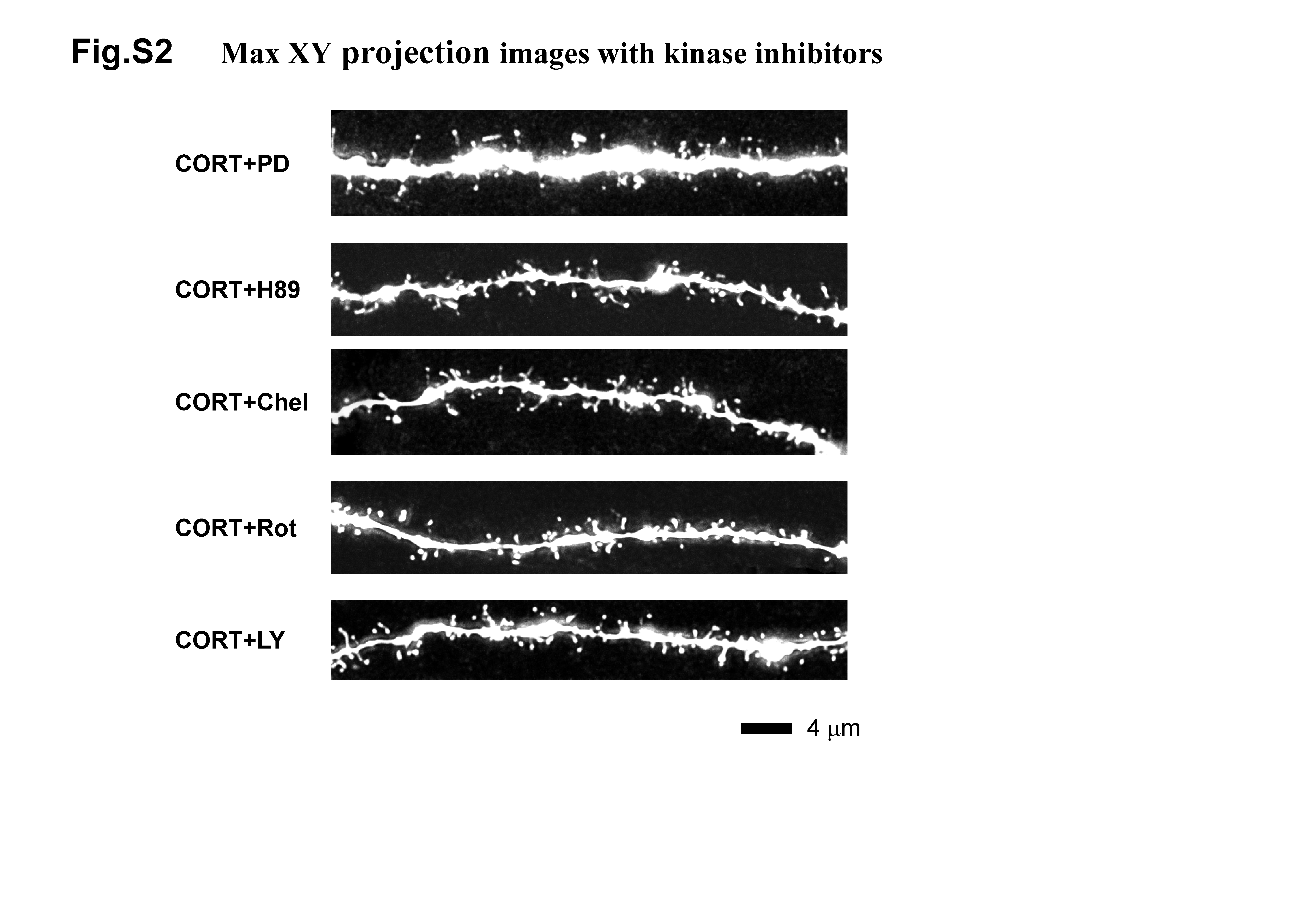

Supplement: Figure S2 — Images for effects by inhibition of kinases on spinogenesis by CORT. Maximal intensity projections onto XY plane from z-series confocal micrographs, showing spines along the secondary dendrites of hippocampal CA1 pyramidal neurons. Dendrites were treated with 1 µM CORT and 20 µM PD98059 (CORT + PD), with 1 µM CORT and 10 µM H-89 (CORT + H89), with 1 µM CORT and 10 µM chelerythrine (CORT + Chel), with 1 µM CORT and 10 µM rottlerin (CORT + Rot), and with 1 µM CORT and 10 µM LY294002 (CORT + LY) for 1 h. Bar, 4 µm. (TIFF) [file pone.0034124.s002.tiff]

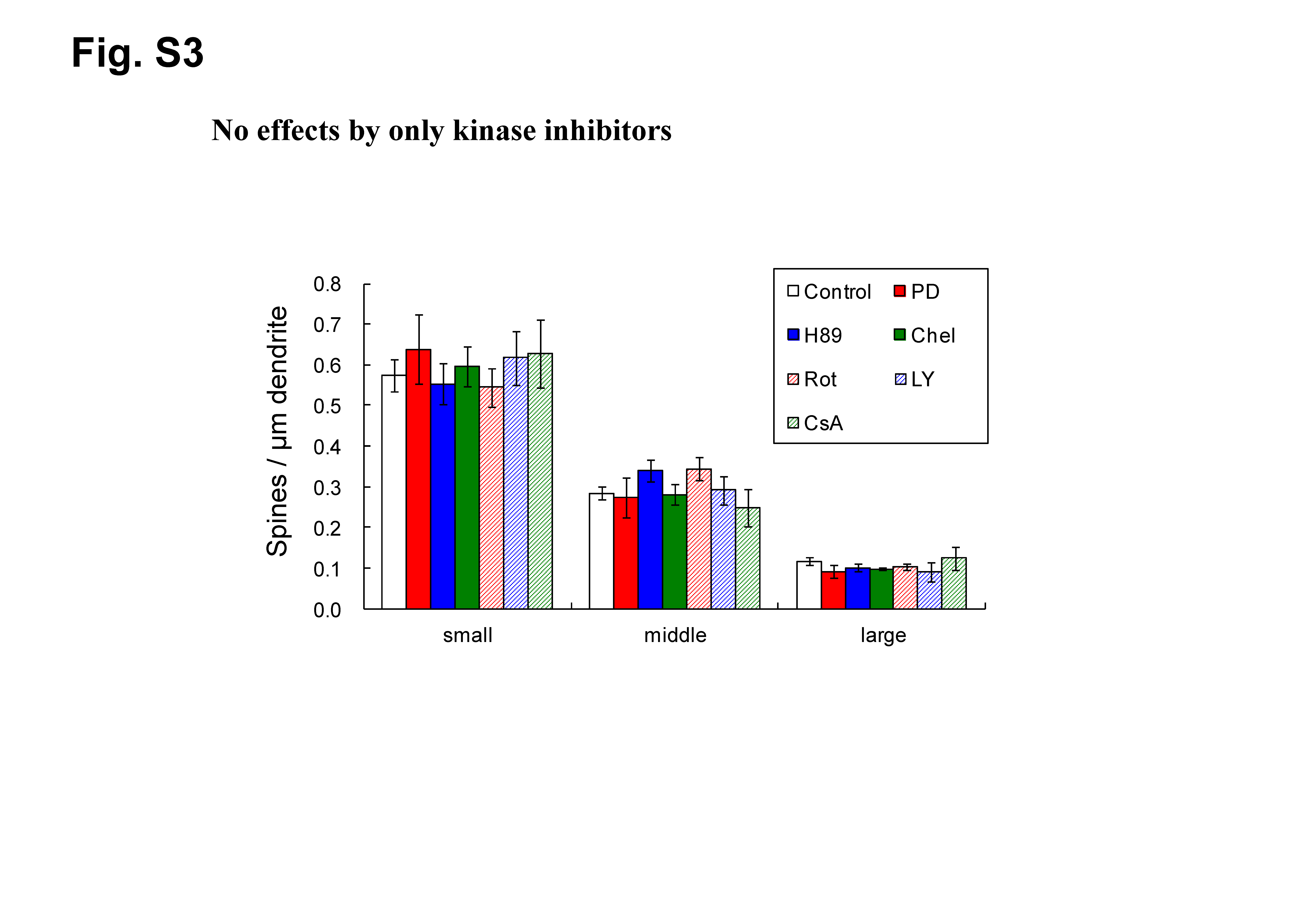

Supplement: Figure S3 — No effect of kinase inhibitors alone on the density of three subtypes of spines in CA1 neurons. A 1 h treatment in ACSF without drugs (Control, open column), with 20 µM PD98059 (PD, red column), with 10 µM H-89 (H89, blue column), with 10 µM chelerythrine (Chel, green column), with 10 µM rottlerin (Rot, hatched red column), with 10 µM LY294002 (LY, hatched blue column), and with 1 µM cyclosporin A (CsA, hatched green column). From left to right, small-head spines (small), middle-head spines (middle), and large-head spines (large). Vertical axis is the average number of spines per 1 µm of dendrite. Statistically significant differences were not observed upon inhibitor alone treatment. Results are reported as mean ± SEM. For each drug treatment, we investigated 3 rats, 6 slices, 12 neurons, 24 dendrites and 1200–1500 spines. The significance of CORT or drug effect was examined using the Tukey–Kramer post hoc multiple comparisons test when one way ANOVA tests yielded P<0.05. (TIFF) [file pone.0034124.s003.tiff]

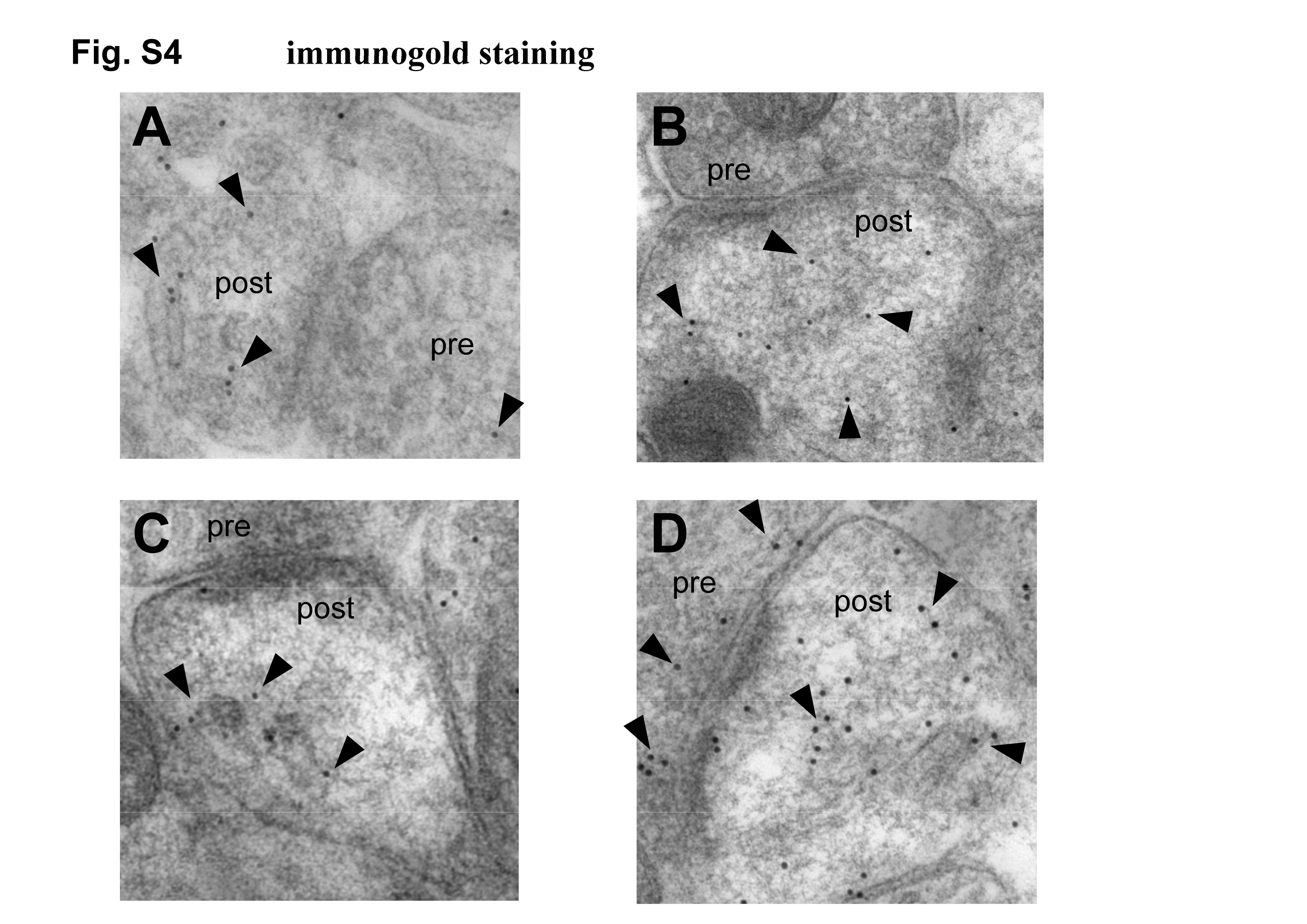

Supplement: Figure S4 — Immunoelectron microscopic analysis of the distribution of GR within the axospinous synapses in the stratum radiatum of the hippocampal principal neurons of the CA1 (A)–(D). Gold particles (arrowheads), specifically indicating the presence of GR, were localized in the presynaptic and postsynaptic regions. In dendritic spines, gold particles were found within the spine head and, in some cases were associated with PSD regions, axospinous synapses of the hippocampal principal neurons in the stratum radiatum of the CA1. A search for immunogold-labeled GR proteins was performed for at least 30 synapses at the CA1 region from more than 100 independent images. A 1∶1000 dilution of antiserum was used to prevent nonspecific labeling. Preadsorption of the antibody with GR antigen (30 µg/ml) resulted in the disappearance of immunoreactivity. Pre, presynaptic region; post, postsynaptic region. Scale bar, 200 nm. (TIF) [file pone.0034124.s004.tif]

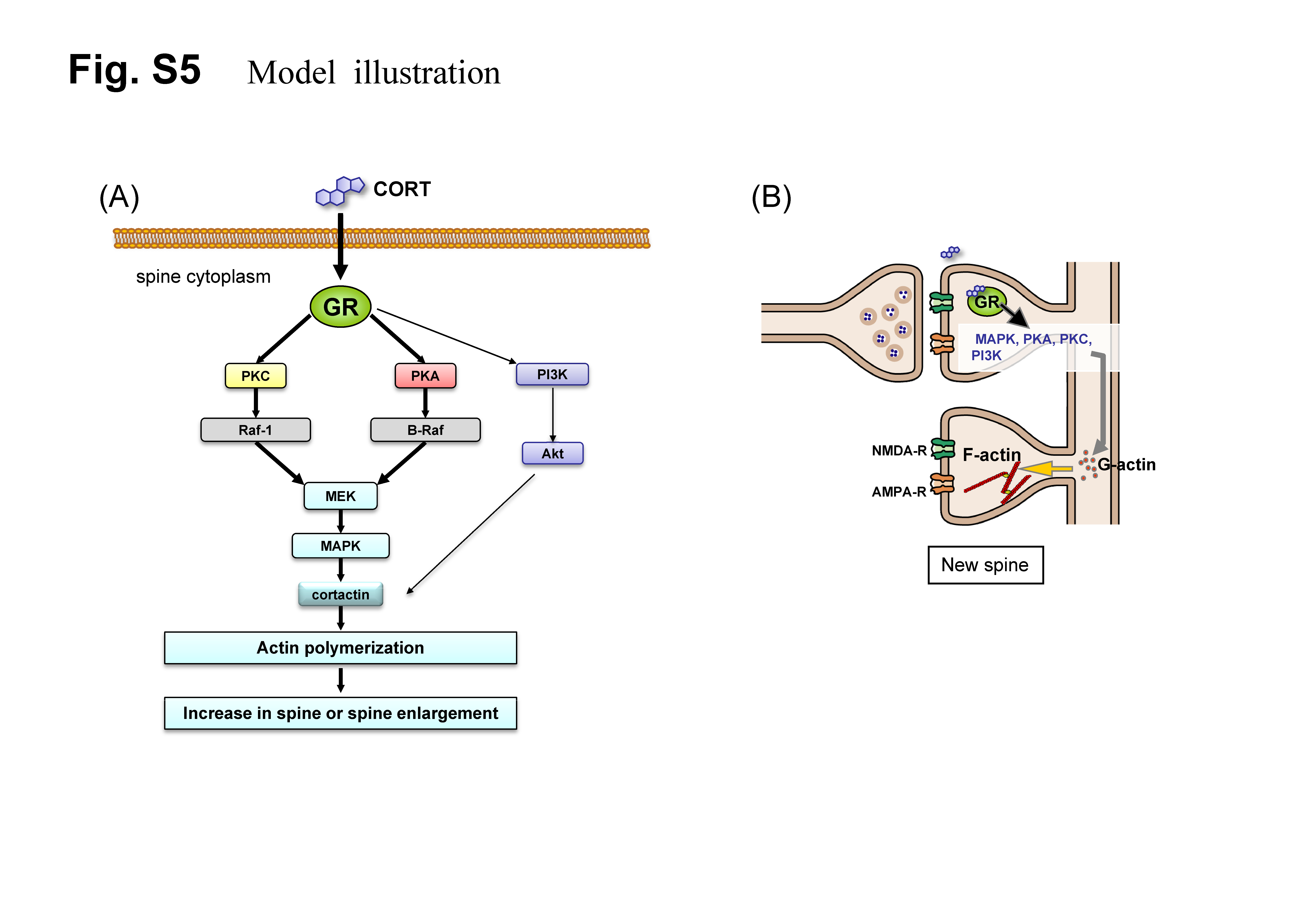

Supplement: Figure S5 — Model illustration. (A) Schematic illustration of CORT-driven multiple kinase pathways. Upon binding of CORT, GR induces the sequential activation of PKA, PKC, MEK, and Erk MAPK. Erk MAPK regulates phosphorylation of actin-related proteins such as cortactin, resulting in actin reorganization. (B) Schematic illustration of CORT-induced rapid spinogenesis via multiple kinase pathways. (TIFF) [file pone.0034124.s005.tiff]

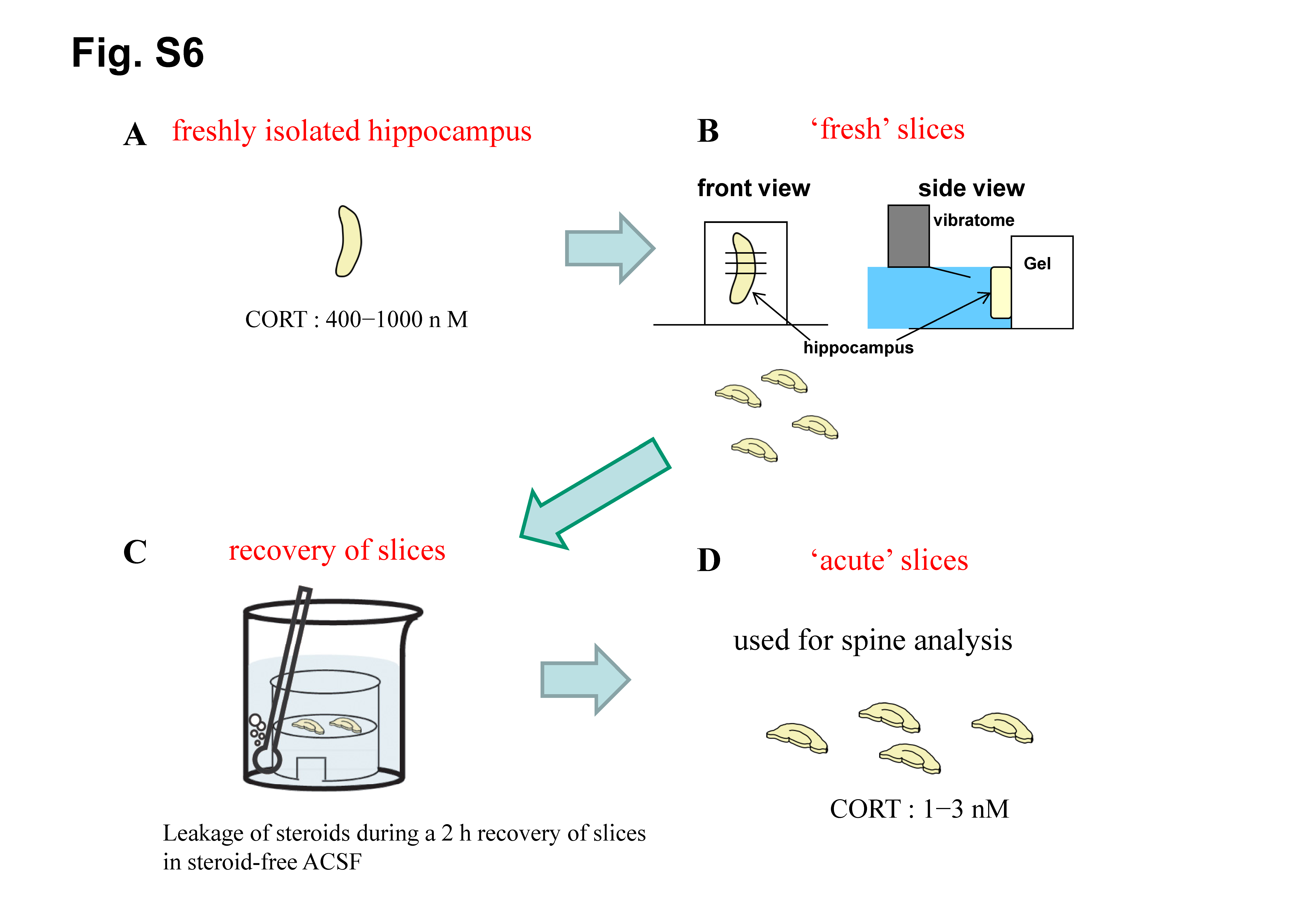

Supplement: Figure S6 — Depletion of CORT and other sex-steroids occurs by ACSF incubation to obtain ‘acute’ slices which are used widely for investigations of spinogenesis and electrophysiology. (TIFF) [file pone.0034124.s006.tiff]

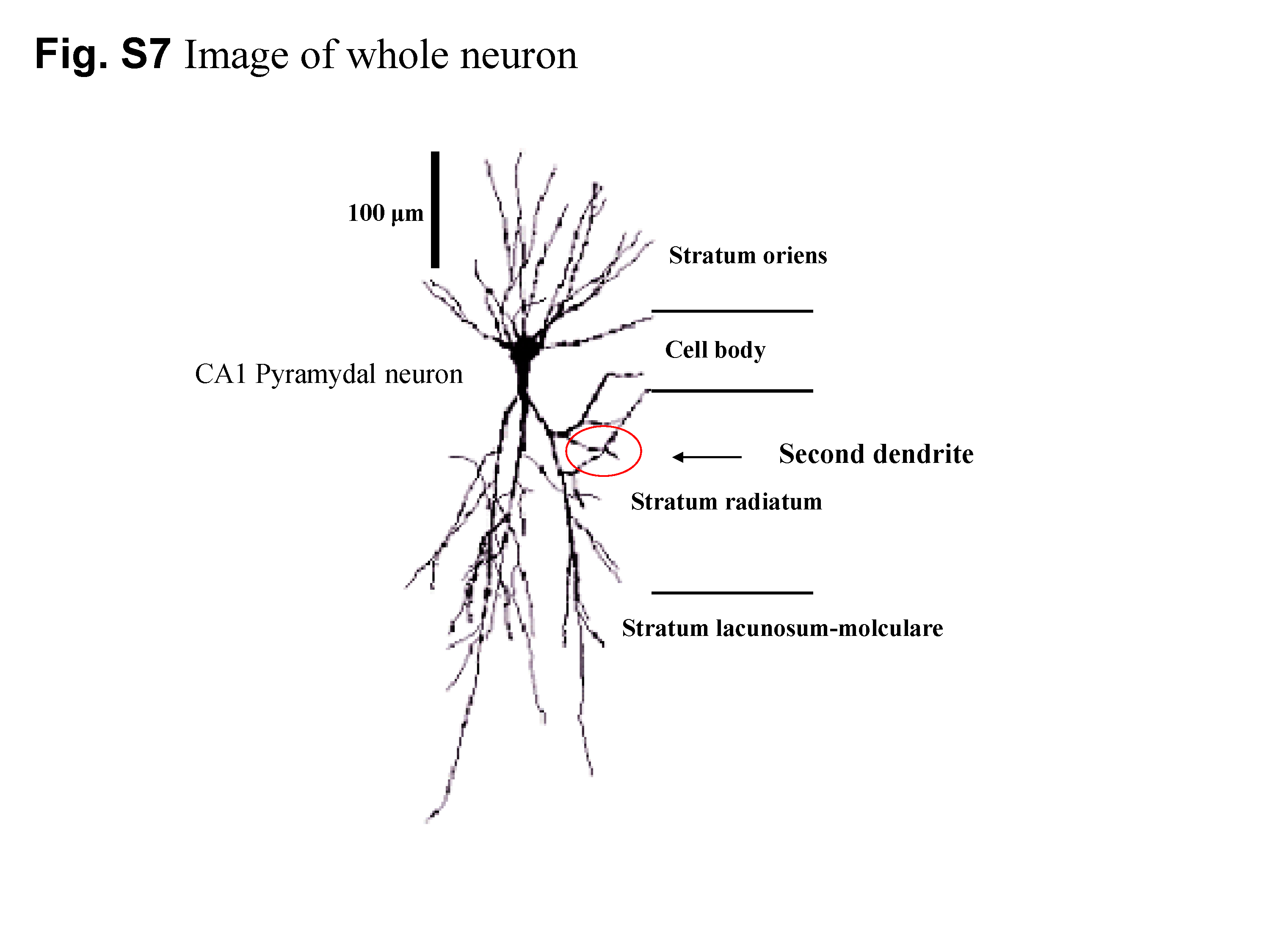

Supplement: Figure S7 — A confocal micrograph showing Lucifer Yellow-injected whole neuron morphology including cell body and dendrites of hippocampal CA1 pyramidal neurons. No significant changes were observed in whole neuron shape and dendrite structures before and after CORT application for 1 h. Secondary dendritic spines have been shown to be highly sensitive to sex-steroid hormones from many early studies. Vertical bar 100 µm. (TIFF) [file pone.0034124.s007.tiff]
