# Supplementary material for: Corticosterone Induces Rapid Spinogenesis via Synaptic Glucocorticoid Receptors and Kinase Networks in Hippocampus
Source: PLoS One. 2012 Apr 11;7(4):e34124. doi: 10.1371/journal.pone.0034124 (PMC3324490; doi:10.1371/journal.pone.0034124)
Supplement: Text S1 — Supporting information for materials and methods, and results. (DOC) [file pone.0034124.s008.doc]

# Supporting Information

## Materials and Methods

##### Postembedding immunogold method for electron microscopy

Immunoelectroscopic analysis was performed essentially as described elsewhere [1,2]. Rat hippocampus was frozen and sliced coronally. Freeze substitution and low-temperature embedding of the specimens was performed as described previously [3]. The samples were immersed in uranyl acetate in anhydrous methanol (-90 ºC). The samples were infiltrated with Lowicryl HM20 resin (Electron Microscopy Sciences, USA) and polymerization was performed with ultraviolet light. Ultrathin sections were cut using a Reichert-Jung ultramicrotome. For immunolabeling, sections were incubated with primary antibody for GR (a gift from Prof. M. Kawata) (diluted to 1/3000) overnight, and incubated with secondary gold-tagged (10 nm) Fab fragment in Tris buffered saline (TBS). Sections were counterstained with 1% uranyl acetate, and viewed on a JEOL 1200EX electron microscope (Japan). Images were captured using a CCD camera (Advanced Microscopy Techniques, USA).

### Mass-spectrometric assay of CORT

Detailed procedures of LC-MS/MS are described elsewhere [2,4]. The extraction of steroids from hippocampal slices was performed by a hexane : ethylacetate = 2 : 3 mixture. The steroid extracts were applied to a C_18_ Amprep solid phase column (Amersham Biosciences, USA). The CORT fraction was purified from the eluted steroids using a normal phase HPLC system (Jasco, Japan) with a silica gel column. The same slices as those used for spine analysis were homogenized, and steroid extraction was performed. The LC-MS/MS system, which consisted of a reverse phase LC coupled with an API 5000 triple-stage quadrupole mass spectrometer (Applied Biosystems, USA), was operated with electron spray ionization in the positive-ion mode. The LC chromatographic separation for CORT was performed on a Cadenza CD-C_18_ column (Imtakt, Japan). The MS/MS process monitored the m/z transition from 347 to 121 for CORT. d_8_-CORT was used as internal standards in order to measure the recovery of CORT and to calibrate the retention time. Chromatographic profiles for the fragmented ion of CORT with m/z = 121 showed a clear peak with a retention time of 5.75 min, which was the same retention time obtained for the fragmented ion of standard CORT.

## Results

### Western blot analysis of GR in synaptic and extranuclear fractions

Extended analysis indicated that some GRs was localized in the purified PSD fraction, which was characterized by PSD-95, as described [2]. GR was observed in cytoplasmic fraction (Cyt), presynaptic membrane-rich fraction (PRE, characterized by synaptophysin), high density membrane fraction (HDM, containing microsomes), and PSD fraction (PSD). The total integrated amount of GR was largest in the Cyt fraction, relative to other fractions. Reactivity of the GR antibody has been verified with Western blot using synthestic GR protein, liver and hippocampus [5,6]. The hippocampal homogenates have the same GR expression at 97 kD as that in liver (positive control) [5].

### Ultrastructural analysis for synaptic and nuclear localization of GR

To explain the site of rapid spinogenesis by the activation of GR, a clarification of the synaptic localization of GR in glutamatergic neurons is essential. The extranuclear and nuclear localization of GR was clarified via ultrastructural investigations using GR IgG (1/3000). An immunoelectron microscopic analysis using post-embedded immunogold was performed to determine the localization of GR-immunoreactivity in the hippocampal CA1 neurons of adult male rats. GR was localized not only in the nuclei but also in both the axon terminals and dendritic spines of principal neurons (Fig. S4) [2]. Gold particles were clustered in the postsynaptic and presynaptic compartments, as well as the nuclei. At postsynapses, gold particles were distributed within the cytoplasm of the spine head. In some cases, gold particles were affiliated within the postsynaptic density. In glial-like cells, gold particles were not clearly recognized. To ensure specific labeling, multiple labeling (3 or more) of immunogold in the pre- and post-synaptic compartments was confirmed. For a search of immunogold-labeled GR proteins, we used at least 100 images. Each image contained several synapses among which at least 1 synapse expressed GR particles. We also observed some synapses in one image that did not express GR particles. Consequently, we observed roughly 25-30 % of synapses that expressed GR particles. Preadsorption of the antibody with GR antigen (30 μg/ml) resulted in the disappearance of immunoreactivity.

### Depletion of CORT in ‘acute’ slices (Fig. S6)

The CORT concentration in the hippocampal slices was determined before and after 2 h incubation with steroid-free ACSF, by mass-spectrometric analysis [2]. After a 2 h incubation in steroid-free ACSF, the CORT level was approx. 2 nM (‘acute’ slices used for spine analysis), however, the CORT level was 200-800 nM before ACSF incubation (‘fresh’ hippocampal slices). These results indicated that a significant release of CORT into ACSF occurred during incubation for 2 h.

## References

1. Mukai H, Tsurugizawa T, Murakami G, Kominami S, Ishii H, et al. (2007) Rapid modulation of long-term depression and spinogenesis via synaptic estrogen receptors in hippocampal principal neurons. J Neurochem 100: 950-967.

2. Ooishi Y, Mukai H, Hojo Y, Murakami G, Hasegawa Y, et al. (2011) Estradiol rapidly rescues synaptic transmission from corticosterone-induced suppression via synaptic/extranuclear steroid receptors in the hippocampus. Cerebral Cortex Web online.

3. Roberson ED, English JD, Adams JP, Selcher JC, Kondratick C, et al. (1999) The mitogen-activated protein kinase cascade couples PKA and PKC to cAMP response element binding protein phosphorylation in area CA1 of hippocampus. J Neurosci 19: 4337-4348.

4. Hojo Y, Higo S, Ishii H, Ooishi Y, Mukai H, et al. (2009) Comparison between hippocampus-synthesized and circulation-derived sex steroids in the hippocampus. Endocrinology 150: 5106-5112.

5. Komatsuzaki Y, Murakami G, Tsurugizawa T, Mukai H, Tanabe N, et al. (2005) Rapid spinogenesis of pyramidal neurons induced by activation of glucocorticoid receptors in adult male rat hippocampus. Biochem Biophys Res Commun 335: 1002-1007.

6. Morimoto M, Morita N, Ozawa H, Yokoyama K, Kawata M (1996) Distribution of glucocorticoid receptor immunoreactivity and mRNA in the rat brain: an immunohistochemical and in situ hybridization study. Neurosci Res 26: 235-269.
